# Distributed Medical Education (DME) in psychiatry: perspectives on facilitators, obstacles, and factors affecting psychiatrists' willingness to engage in teaching activities

**DOI:** 10.1186/s12909-024-05178-8

**Published:** 2024-02-25

**Authors:** Raquel da Luz Dias, Lara Hazelton, Mandy Esliger, Peggy Alexiadis Brown, Philip G. Tibbo, Nachiketa Sinha, Anthony Njoku, Satyanarayana Satyendra, Sanjay Siddhartha, Faisal Rahman, Hugh Maguire, Gerald Gray, Mark Bosma, Deborah Parker, Owen Connolly, Adewale Raji, Alexandra Manning, Alexa Bagnell, Reham Shalaby, Vincent Israel Opoku Agyapong

**Affiliations:** 1 Department of Psychiatry, Faculty of Medicine, Dalhousie University, Halifax, NS Canada; 2 Department of Psychiatry, Faculty of Medicine, Dalhousie University, Saint John, NB Canada; 3 Department of Psychiatry, Faculty of Dentistry and Medicine, University of Alberta, Edmonton, AB Canada

**Keywords:** Distributed medical education, Continued medical education, Medical residency, Rural health services, Psychiatry

## Abstract

**Background:**

Distributed Medical Education (DME), a decentralized model focused on smaller cities and communities, has been implemented worldwide to bridge the gap in psychiatric education. Faculty engagement in teaching activities such as clinical teaching, supervision, and examinations is a crucial aspect of DME sites. Implementing or expanding DME sites requires careful consideration to identify enablers that contribute to success and barriers that need to be addressed. This study aims to examine enablers, barriers, and factors influencing psychiatrists' willingness to start or continue participating in teaching activities within Dalhousie University's Faculty of Medicine DME sites in two provinces in Atlantic Canada.

**Methodology:**

This cross-sectional study was conducted as part of an environmental scan of Dalhousie Faculty of Medicine’s DME programs in Nova Scotia (NS) and New Brunswick (NB), Canada. In February 2023, psychiatrists from seven administrative health zones in these provinces anonymously participated in an online survey. The survey, created with OPINIO, collected data on sociodemographic factors, practice-related characteristics, medical education, and barriers to teaching activities. Five key outcomes were assessed, which included psychiatrists' willingness to engage in (i) clinical training and supervision, (ii) lectures or skills-based teaching, (iii) skills-based examinations, (iv) training and supervision of Canadian-trained psychiatrists, and (v) training and supervision of internationally trained psychiatrists. The study employed various statistical analyses, including descriptive analysis, chi-square tests, and logistic regression, to identify potential predictors associated with each outcome variable.

**Results:**

The study involved 60 psychiatrists, primarily male (69%), practicing in NS (53.3%), with international medical education (69%), mainly working in outpatient services (41%). Notably, 60.3% lacked formal medical education training, yet they did not perceive the lack of training as a significant barrier, but lack of protected time as the main one. Despite this, there was a strong willingness to engage in teaching activities, with an average positive response rate of 81.98%. The lack of protected time for teaching/training was a major barrier reported by study participants. Availability to take the Royal College of Physicians and Surgeons of Canada Competency by Design training was the main factor associated with psychiatrists' willingness to participate in the five teaching activities investigated in this study: willingness to participate in clinical training and supervision of psychiatry residents (*p* = .01); provision of lectures or skills-based teaching for psychiatry residents (*p* < .01); skills-based examinations of psychiatry residents (*p* < .001); training/supervision of Canadian-trained psychiatrists (*p* < .01); and training and supervision of internationally trained psychiatrists (*p* < .01).

**Conclusion:**

The study reveals a nuanced picture regarding psychiatrists' engagement in teaching activities at DME sites. Despite a significant association between interest in formal medical education training and willingness to participate in teaching activities, clinicians do not consider the lack of formal training as a barrier. Addressing this complexity requires thoughtful strategies, potentially involving resource allocation, policy modifications, and adjustments to incentive structures by relevant institutions.

**Supplementary Information:**

The online version contains supplementary material available at 10.1186/s12909-024-05178-8.

## Background

The increasing global recognition of mental health as a prominent public health concern has highlighted the need for medical students to receive comprehensive training in psychiatry to effectively address the growing burden of mental health disorders worldwide [1]. However, the conventional medical education system, primarily centered in large urban areas, has often failed to adequately meet the healthcare needs of smaller cities and communities [2]. To bridge this gap, Distributed Medical Education (DME), a decentralized model of health education integrated into and accountable to local communities, has been implemented and expanded across Canada and other parts of the world [3]. This approach to medical education emerged in the 1970’s as a dynamic solution to address the evolving requirements of healthcare training and delivery by dispersing educational activities across multiple sites, aiming to enhance access, promote regional healthcare equity, and foster a diverse healthcare workforce [2]. In the Canadian context, the integration of DME into undergraduate and postgraduate medical education in most medical schools has been a significant advancement in promoting regional healthcare recruitment and retention. Despite these efforts, the distribution of physicians across the country remains imbalanced, with only 9.4% of physicians practicing in rural areas, compared to 21.1% of Canadians residing in rural and small towns [4]. When it comes to the number of specialists, the urban-rural disparity in specialist numbers and distribution becomes evident when examining the physician-to-population ratios for different segments of the Canadian populace. Approximately 30.3% of Canadians reside in rural regions where the specialist-to-population ratios fall within the range of 0.1 to 5.0/10,000, while 24.2% inhabit urban areas where this ratio expands to 15.1 to 30.0/10,000 [5].

The Atlantic Canada, also known as the Maritimes region, closely mirrors national medical education and healthcare challenges. Spanning provinces such as Nova Scotia (NS), New Brunswick (NB), and Prince Edward Island (PEI), this region grapples with unique geographical spread, encompassing urban centers and remote communities. Historically, the Maritimes have been characterized by a strong sense of community, a close-knit healthcare network, and approaches to medical training that aim to meet the specific needs and aspirations of both learners and educators. Dalhousie University, a pivotal institution in the region, operates in two primary campuses. The Dalhousie Medical School, located in Halifax, NS, has been a cornerstone of medical education for nearly 150 years since its establishment in 1868. Building on this legacy, Dalhousie expanded its reach with the establishment of Dalhousie Medicine New Brunswick in 2010, situated in Saint John, NB. A third distributed campus is currently in the planning phase and is expected to admit students in Cape Breton, NS, by 2025. Traditionally, psychiatry residency training has been offered at the Halifax campus, with residents spending shorter amounts of time in other sites outside the main academic center. Recognizing the growing population and the need to fill existing vacancies in the psychiatry residency program, the governments of Nova Scotia (NS) and New Brunswick (NB), which are two of the most populous provinces in the Atlantic region, approached Dalhousie University to request an increase in the number of psychiatry residency training positions. The further increase in training spots would be accomplished through expanding the amount of DME, and might involve developing programs based primarily outside Halifax. The governments’ requests align with the principles underlying the Dalhousie Department of Psychiatry's Transformational Plan [6], which aims to extend its academic mandate by enhancing medical education, research, and knowledge translation activities at distributed learning sites in NS and NB. These coordinated efforts aim to improve psychiatrist recruitment and retention while also meeting the region's healthcare demands.

However, the expansion of DME sites requires careful consideration to identify enablers that contribute to success and barriers that need to be addressed. Faculty engagement plays a pivotal role at distributed sites, and academic departments must effectively engage and integrate distributed faculty into formal teaching activities in a way that is inclusive and respectful of the informal teaching activities that may already exist. It is crucial to avoid pitfalls such as insufficient engagement, unclear expectations, and resistance to change. Conversely, clear strategic planning and collaborative governance involving provincial health authorities are vital factors that must be pursued in this process [6, 7]. Attending to faculty members' perspectives regarding DME may facilitate the adoption of a growth mindset, openness to new approaches, and active participation in professional development to navigate challenges. By embracing these principles, faculty members can enhance their engagement, promote effective teaching practices, and make valuable contributions to the success of DME programs [8].

While there is existing literature on Distributed Medical Education (DME) in both Canadian and international contexts, there is a notable gap in understanding psychiatry education within distributed medical sites. Specifically, there is limited knowledge about the factors influencing psychiatrists' willingness to participate in teaching activities, which is crucial for the effective implementation and expansion of DME. This study aims to bridge this gap by investigating the enablers, barriers, and other factors that impact psychiatrists' willingness to engage in teaching activities, a key component of DME expansion. The objective is to deepen our understanding of how psychiatrists perceive DME, facilitating more informed decisions regarding the expansion of DME within Dalhousie University's Department of Psychiatry. The insights gained from this study have the potential to contribute significantly to the ongoing conversation surrounding DME in Atlantic Canada and beyond.

## Methods

### Study design

This cross-sectional study was part of an environmental scan completed at the Dalhousie Faculty of Medicine’s Department of Psychiatry distributed education sites in NS and NB. The environmental scan adopted a formal information search approach and an explanatory design, including quantitative and qualitative data, collected through online surveys and focus groups meetings, respectively [9]. This study included a dataset of the quantitative data, focused on teaching activities and variables related to that.

### Study settings and participants

This study involved psychiatrists from seven administrative health zones (three in NS and four in NB) engaged in medical education in distributed sites. The DME sites affiliated with Dalhousie University Faculty of Medicine include mental health and addiction treatment facilities in Nova Scotia's Eastern, Western, and Northern zones, as well as four health zones in New Brunswick. Respondents from different sites, from small rural communities to medium-sized regional centers were included in the study, ensuring representation of the various DME sites in the two Provinces. Given the exploratory and descriptive nature of the environmental scan, the emphasis might have been on understanding various factors influencing psychiatrists' willingness to engage in teaching activities rather than on achieving a predetermined statistical power.

### Study procedures and data collection

Members of the Nova Scotia and New Brunswick Psychiatry Academic Council developed and revised an online survey using OPINIO [10]. The survey was then distributed to all psychiatrists working within the seven DME administrative health zones via their clinical department heads. To maximize the response rate, psychiatrists were invited by their clinical department heads to complete the surveys during monthly psychiatrists' meetings for each zone as a group activity. Each psychiatrist was given time to complete the survey anonymously on their personal cell phone or computer. For those unable to attend the meeting, a second electronic reminder was sent to ensure they completed the survey. Data collection took place between January and February 2023. Respondents were informed that survey completion was voluntary and that the data collected would be used for research purposes.

### Outcome measures

**Fig. 1:**
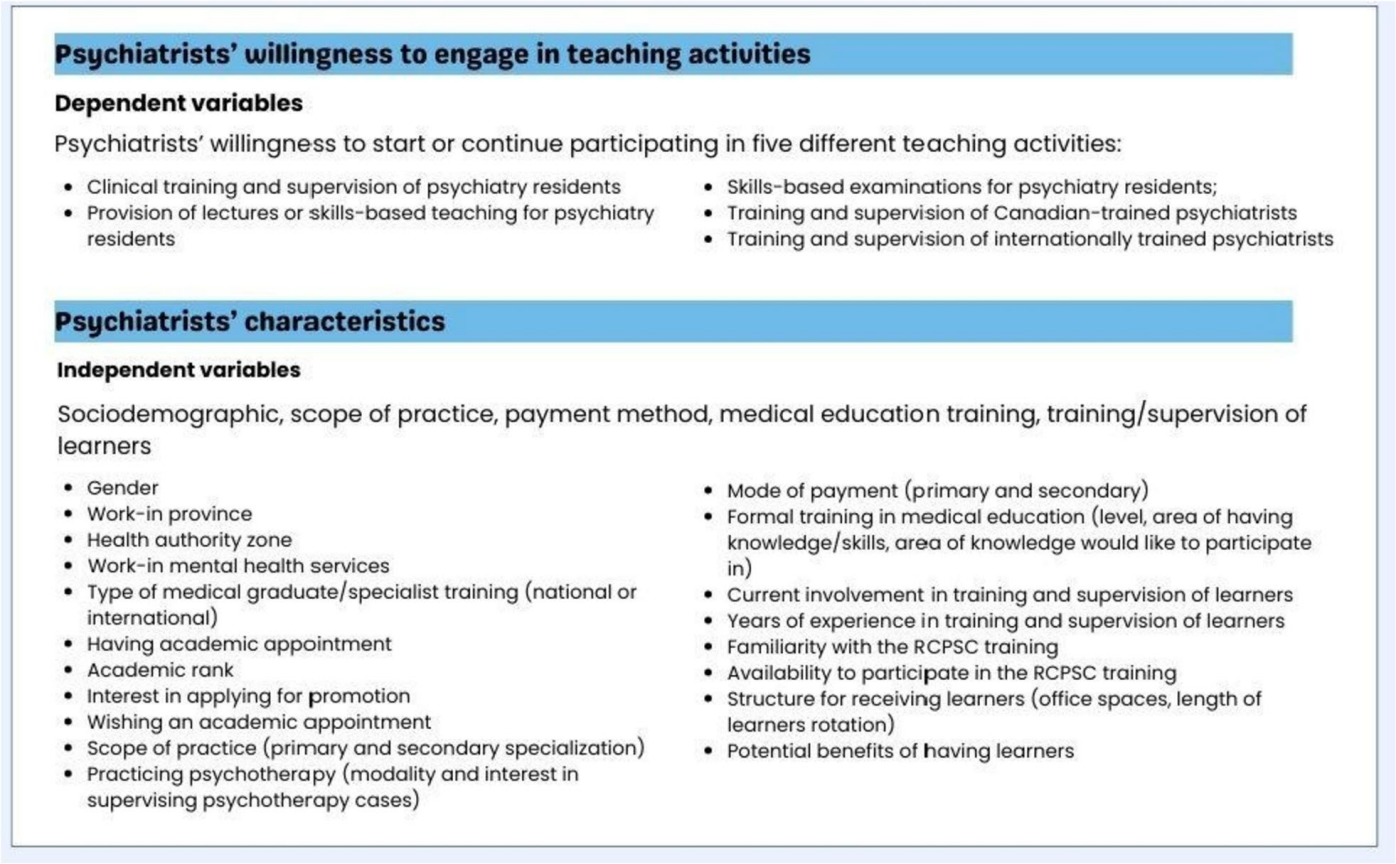
Outcomes of interest

The outcomes of interest (Fig. 1) comprised frequency distribution of psychiatrists' characteristics, including sociodemographic, scope of practice, payment methods, medical education training, and involvement in training/supervision, barriers to receiving trainees, and their willingness to participate in five distinct teaching activities. The inclusion of the selected variables in the study was driven by the comprehensive nature of this investigation, aiming to capture a holistic view of the factors influencing psychiatrists' willingness to engage in teaching activities, and recognizing that these variables collectively contribute to the intricate landscape of physicians' professional lives (i.e. career planning, ability to travel to and from distributed sites, workload, etc.). Utilizing correlational, association, and regression analyses, we investigate the relationship between psychiatrists' willingness to engage in teaching (considered dependent variables) and their diverse characteristics (considered independent variables). These analyses aimed to identify potential significant predictors for each of the specified outcome variables, allowing for a more informed and context-rich exploration of the research question.

### Statistical analysis

Results were analyzed using SPSS version 28 [11]. Descriptive analysis was performed for all the variables in this study, data were summarized and reported as frequency distribution using numbers and percentages. All study data were grouped into seven main categories; participants’ sociodemographic characteristics, practice-related variables, payment-related variables, medical education training variables, involvement with training/supervision related variables, barriers to receive trainees, and willingness to participate in teaching activities variables. Chi square or the Fischer Exact test with two-tailed significance (p ≤ 0.05) was used to define the distribution of selected categorical variables against the five study outcome variables.

Five models of multivariate binary logistic regression analyses were employed to identify potential significant predictors for each outcome variable, while controlling for other variables. Fulfilling all the assumptions of this type of analysis, we determined the dependent variables of interest as to be dichotomous outcome variables with Yes/No responses. The independent variables could be either continuous (i.e., an interval or ratio variable) or categorical variables. Additionally, observations were independent, and the dependent variable had mutually exclusive categories. Furthermore, prior to running the analysis, the potential independent variables were examined as to be related to the outcome variables. Variables that showed either significance (p ≤ 0.05) or near significance (0.05 < p < 0.2) obtained from the univariate Chi-squared analysis, were included into the respective regression model. This analysis aimed to capture trends or associations that, while not meeting the conventional threshold for statistical significance, might still indicate potential meaningful relationships. As a variety of factors associated the outcomes of interest are investigated in this study, this approach allows for a more comprehensive understanding of the data. Although it does not provide definitive evidence, it offers a suggestive signal, contributing to a nuanced perspective that acknowledges the complex nature of the studied phenomena under study. Odds ratios (OR) and confidence intervals were reported to determine whether a particular variable is an associative factor for each study outcome, controlling for the rest of the variables. Correlational analysis was performed prior to the regression analyses, to exclude any strong intercorrelations to avoid redundancy (Spearman’s correlation coefficient of 0.7 to 1.0 or − 0.7 to − 1.0) among predictor variables. There was no imputation of missing data and only complete responses were reported.

### Ethics considerations

This study has been developed and executed in accordance with the Declaration of Helsinki for research involving human participants, and was submitted to the Dalhousie University Research Ethics Board. The Board conceived an exemption with the justification that the project is considered a program evaluation/quality assurance and quality improvement activity, a type of study which is exempted from REB review as per the Tri-Council Policy Statement Ethical Conduct for Research Involving Humans article 2.5 [12]. Survey respondents were informed that survey completion was voluntary and anonymous, and that data collected would be used for research purposes. Informed consent was obtained from all participants and consent was implied upon survey completion and submission.

## Results

The study respondents included 60 psychiatrists out of a total of 120 psychiatrists in NS and NB practicing outside of Nova Scotia’s Central Zone representing a survey response rate of 50%.

### Frequency distribution results

**Fig. 2:**
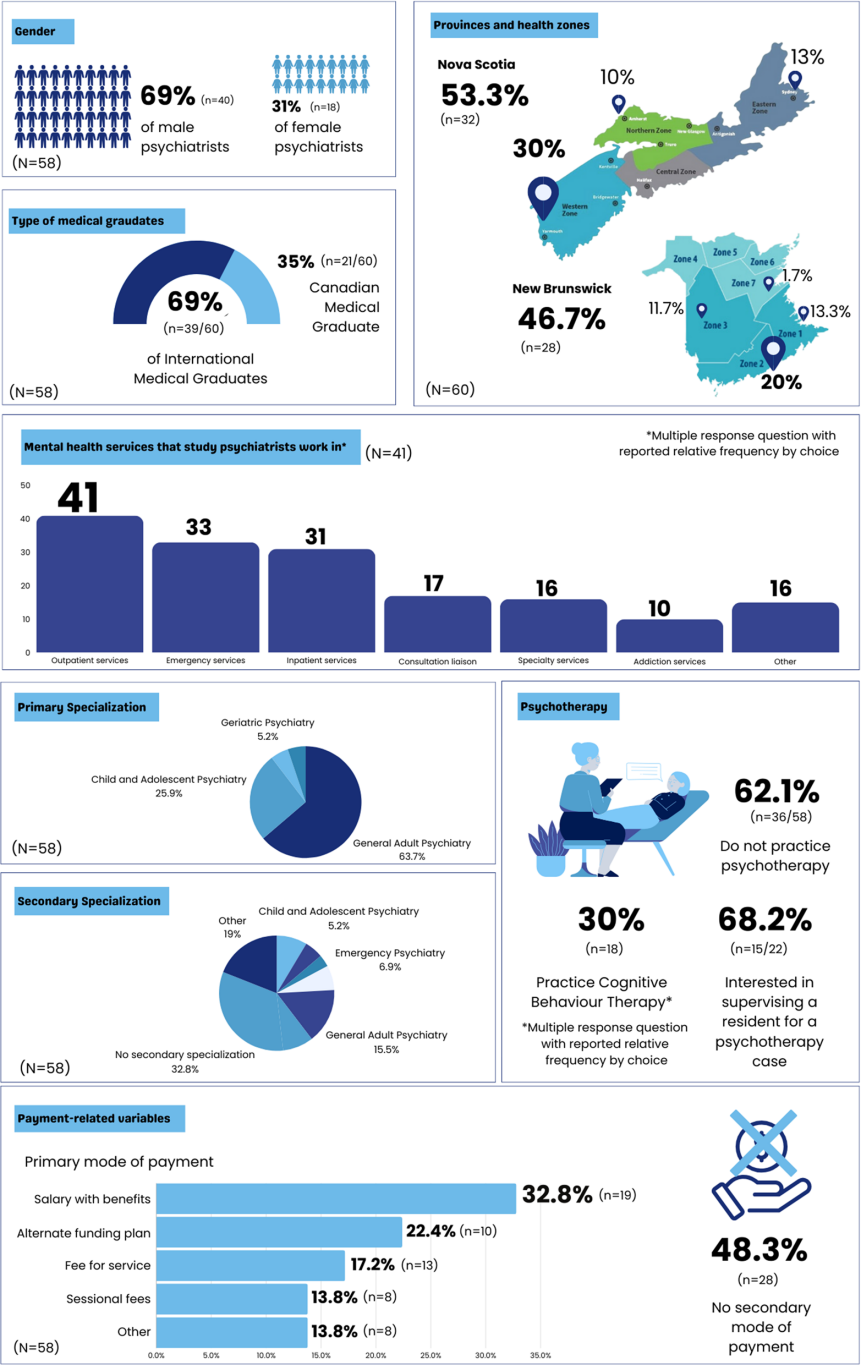
Frequency distribution of psychiatrists’ characteristics (sociodemographic, scope of practice, medical education training and payment methods)

Frequency distribution of the sociodemographic characteristics, scope of practice, medical education training and payment methods is demonstrated in Fig. 2.

Table 1 provides an overview of participants' medical education training, training-related variables, and structural factors. Among the findings, a significant portion of participants (60.3%) did not have formal medical education training. The majority demonstrated knowledge and skills in curriculum delivery and expressed interest in participating in such activities. In terms of training, most participants engaged in clinical training for medical students, with over half having over ten years of experience in this role. A substantial proportion (81.8%) was familiar with the Royal College of Physicians and Surgeons of Canada's Competency by Design (RCPSC/CBD) for residency training and were available for this formal training (76.4%). Infrastructure-wise, most participants felt moderately to slightly adequate, emphasizing specific needs like resident office space and interview rooms. Additionally, the majority believed that two to four months was the ideal duration for Psychiatry resident placements.

**Table 1:** Frequency distribution of variables related to medical education training and training/supervision of learners

	N	%
**Received formal training in medical education**		
Yes	23	39.7
No	35	60.3
**Highest level of medical education received, if applicable**		
Bachelor’s degree	2	8.7
Certificate	3	13.0
Graduate diploma	2	8.7
Master’s degree	4	17.4
Other	12	52.2
Area of medical education having knowledge or skills in*		
I do not have knowledge and/or skills in medical education	18	30
Curriculum development (e.g., developing courses/modules, writing objectives)	10	16.7
Curriculum delivery (e.g., virtual teaching, small group teaching, simulation-based teaching)	29	48.3
Assessment (e.g., writing exam questions, writing OSCE cases)	17	28.3
Program evaluation	8	13.3
Other	7	11.7
**Years of experience in clinical training or supervision of medical learners**		
More than 10 years	30	54.5
6-10 years	9	16.4
3-5 years	9	16.4
1-2 years	5	9.1
0 years	2	3.6
**Familiarity with the RCPSC/CBD training**		
Yes	45	81.8
No	10	18.2
**Availability to participate in formal training in the RCPSC/CBD training**		
Yes	42	76.4
No	13	23.6
**Infrastructure and team set up of participants' service for psychiatry resident**		
Extremely adequate	4	7.4
Moderately adequate	16	29.6
Quite adequate	14	25.9
Slightly adequate	16	29.6
Not at all adequate	4	7.4
**Perceived appropriate length of placement for a Psychiatry resident on rotation with their team**		
Four to six months	12	22.2
Two to four months	19	35.2
One to two months	14	25.9
Other, please specify:	9	16.7

**Fig. 3:**
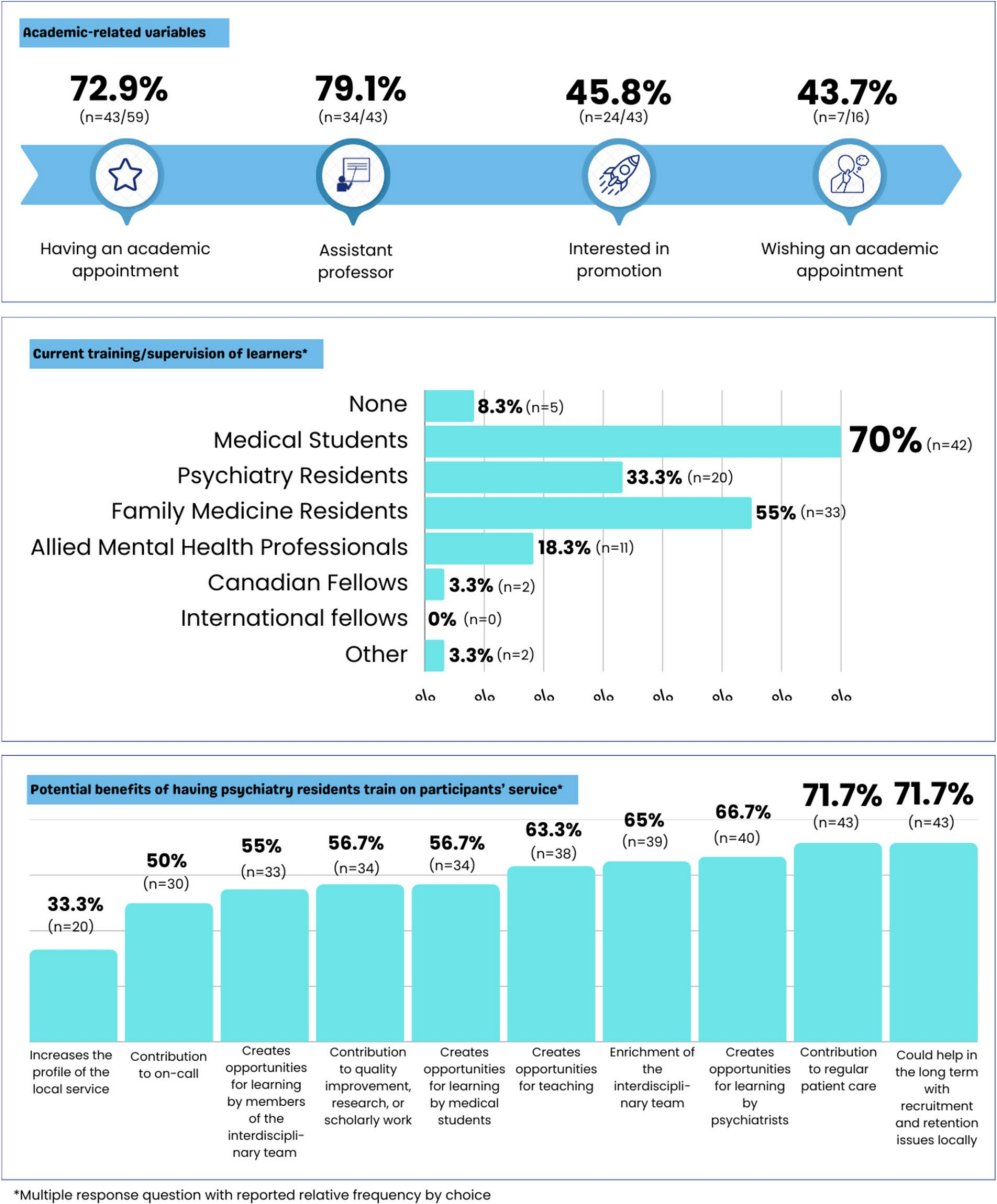
Psychiatrists’ academic position, involvement with training/supervision of learners and perceptions on potential benefits of having trainees in their services

Figure 3 illustrates academic-related characteristics, current involvement in training/supervision of learners, and the perceived benefits of having learners within psychiatrist services. The majority of psychiatrists hold an academic appointment as an assistant professor, and those without it expressed interest in obtaining one. Psychiatry residents ranked third among the categories of learners trained or supervised by participating psychiatrists, following medical students and family medicine residents. While the frequency of training/supervision of psychiatry residents was lower than that for other learner categories, most psychiatrists believed that having psychiatry residents in their services would positively contribute to regular patient care and address local recruitment and retention challenges.

**Fig. 4:**
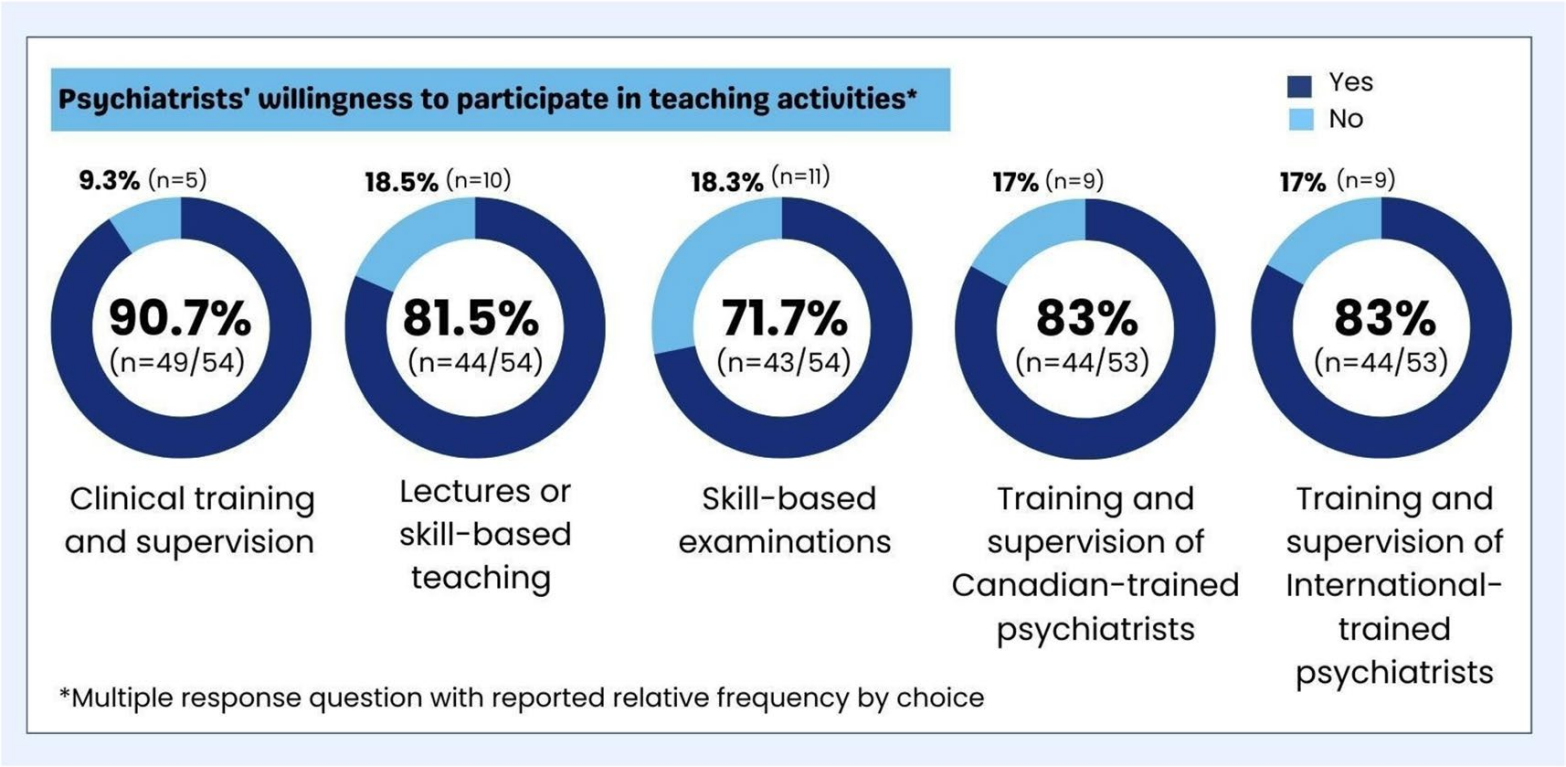
Frequency distribution of key outcomes related to the psychiatrists’ willingness to engage in teaching activities needed to expand DME

Figure 4 displays the frequency distribution of psychiatrists' willingness to engage in five crucial teaching activities for DME expansion. Notably, most psychiatrists were highly interested in all teaching activities with special interest in training and supervising psychiatry residents and both Canadian-trained and internationally-trained psychiatrists within Dalhousie University's one-year accredited fellowship experience.

**Fig. 5:**
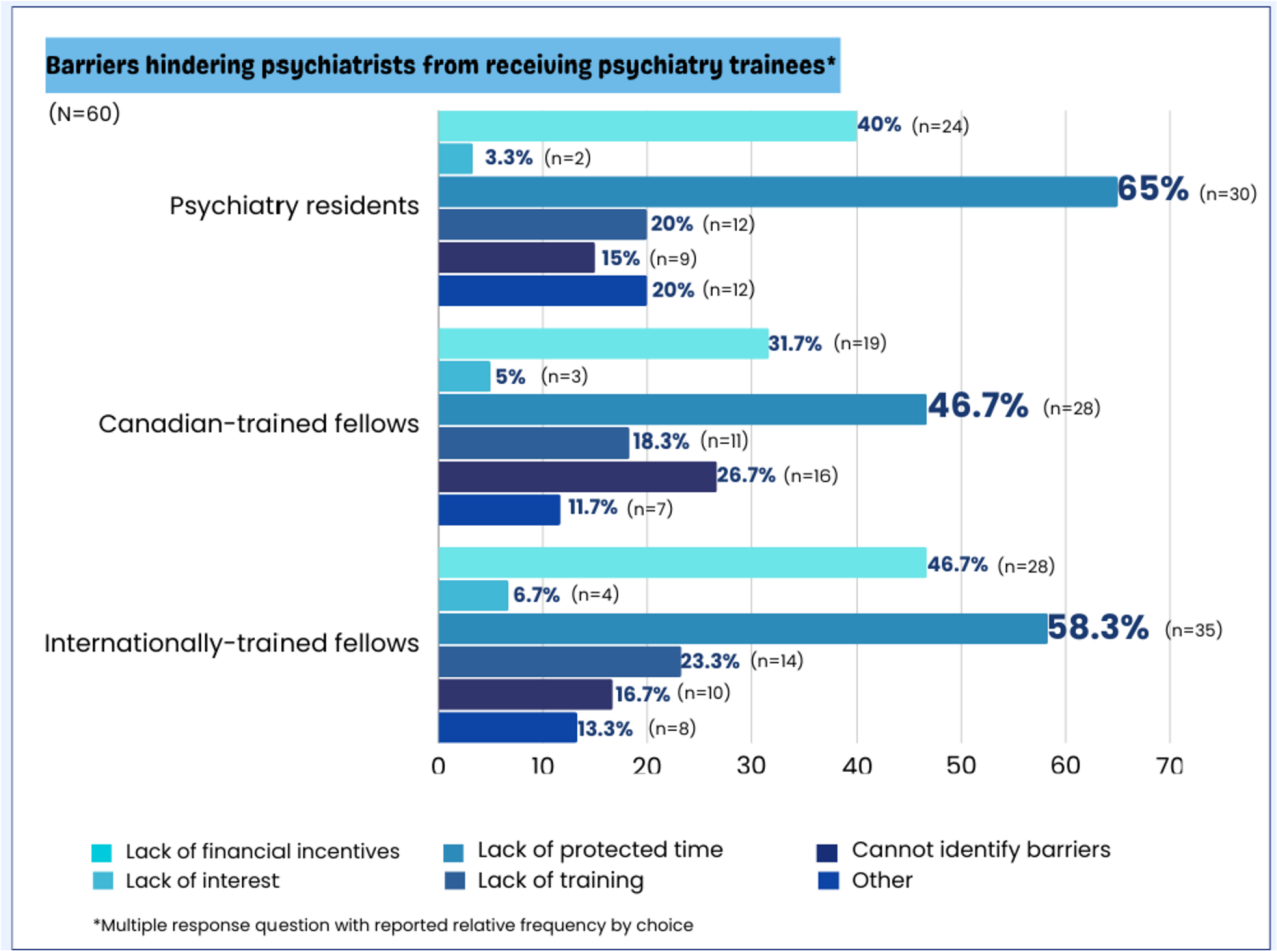
Barriers hindering psychiatrists from receiving psychiatry residents and psychiatry fellows in their practice

Figure 5 demonstrates the barriers hindering psychiatrists from receiving trainees in their practice. The lack of protected time for teaching/training was a major barrier reported by study participants; this impacted survey participants from contributing to the training and supervision of psychiatry residents (65.0%), Canadian (46.7%) and internationally-trained psychiatry fellows (58.3%) on their team.

### Results of association analysis

Table 2 demonstrates statistically significant results from the association analyses of the psychiatrists’ characteristics variables against their willingness to engage in five different teaching activities. It was found that psychiatrists’ ability to train in the Royal College of Physicians and Surgeons of Canada Competency by Design (RCPSC/CBD) training was a significant factor associated with the five outcome variables: willingness to participate in the clinical training and supervision of psychiatry residents (*p* = .01); the provision of lectures or skills-based teaching for psychiatry residents (*p* < .01); skills-based examinations of psychiatry residents (p < .001); training/supervision of Canadian-trained psychiatrists undertaking a one-year Dalhousie University accredited fellowship experience in area of expertise (p < .01); and their willingness to participate in the training and supervision of internationally trained psychiatrists seeking the same fellowship training (*p* < .01).

**Table 2:** Variables presenting statistical significance association with psychiatrists’ willingness to engage in teaching activities

**Variables**	**Clinical training/supervision of psychiatry residents**			**Provision of lectures or skills-based teaching for psychiatry residents**			**Skills-based examinations of psychiatry residents**			**Training/supervision of Canadian-trained psychiatrists**			**Training/supervision of internationally-trained psychiatrists**		
	**N (%)**	**Chi**^**2**^ /Fisher Exact*	***P* value**	**N (%)**	**Chi**^**2**^ /Fisher Exact*	***P* value**	**N (%)**	**Chi**^**2**^ /Fisher Exact*	**P value**	**N (%)**	**Chi**^**2**^ /Fisher Exact*	***P* value**	**N (%)**	**Chi**^**2**^ /Fisher Exact*	***P* value**
**Work-in province**															
Nova Scotia	25 (86.2)	*	.36	20 (69.0)	6.50	**.01**	19 (65.5)	7.69	**<.01**	22 (75.9)	*	.16	22 (75.9)	*	.16
New Brunswick	24 (96.0)			24 (96.0)			24 (96.0)			22 (91.7)			22 (91.7)		
**Having an academic appointment**															
Yes	36 (92.3)	*	.61	35 (89.7)	6.35	**.01**	34 (87.2)	4.93	**.03**	31 (81.6)	0.20	.66	32 (84.2)	0.14	.71
No	13 (86.7)			9 (60.0)			9 (60.0)			13 (86.7)			12 (80.0)		
**Practicing psychotherapy**															
Yes	21 (100.0)	*	.14	20 (95.2)	4.31	**.04**	20 (95.2)	5.16	**.02**	18 (90.0)	1.11	.29	18 (90.0)	1.11	.29
No	28 (84.8)			24 (72.7)			23 (69.7)			26 (78.8)			26 (78.8)		
**Familiarity with the RCPSC/CBDtraining**															
Yes	40 (90.9)	*	.99	37 (84.1)	1.07	.30	38 (86.4)	6.64	**.01**	37 (86.0)	1.48	.22	36 (83.7)	0.08	.78
No	9 (90.0)			7 (70.0)			5 (50.0)			7 (70.0)			8 (80.0)		
**Availability to participate in the RCPSC/CBD training**															
Yes	40 (97.6)	*	**.01**	37 (90.2)	8.67	**<.01**	37 (90.2)	11.83	**<.001**	37 (92.5)	10.40	**<.01**	37 (92.5)	10.40	**<.01**
No	9 (69.2)			7 (53.8)			6 (46.2)			7 (53.8)			7 (53.8)		

Working in New Brunswick, having an academic appointment, and practicing psychotherapy was significantly associated with the psychiatrists’ willingness to participate in the provision of lectures or skills-based teaching and participating in the skills-based examinations of psychiatry residents. Finally, participants who were familiar with the RCPSC training were more willing to start or continue participating in skills-based examinations of psychiatry residents (86.4%), compared to only (50%) of those who were unfamiliar with this training (*p* = .01). Results from the association analysis of other variables that were not statistically significant with the psychiatrists’ willingness to participate in teaching activities are available in the supplementary material section (Table S1 and Table S2).

#### Results of multivariate binary logistic regression analyses

Several variables approached significance with the outcome variables (0.05 < *p* < 0.2) (Table S1 and Table S2). These variables, along with those demonstrating statistical significance (p ≤ .05) in the Chi-squared analyses (Table 2), were included in five logistic regression models, each corresponding to an outcome measure (willingness to participate in five different teaching activities).

Prior to the regression analysis, a correlation analysis revealed high inter-correlation (rs ≥ 0.7) between two variables: Work-in province and work-in Horizon Health Zone. In such cases, the latter variable was omitted from the logistic regression model to avoid redundancy. Similarly, another pair of variables, type of medical graduates and type of completed specialist training, exhibited high inter-correlation, leading to the removal of the first variable from the corresponding logistic regression model whenever both were eligible candidates for regression analysis. The five regression models produced the following results (detailed regression models are available as appendix in the supplementary material section):

1. The model predicting the *willingness to start or continue participating in clinical training and supervision of psychiatry residents* was statistically significant; *Χ*^*2*^ (df=2; *n*=54) = 11.63, *p* < .01, accounting for 19.4% (Cox and Snell R^2^) to 42.1% (Nagelkerke R^2^) of the variance; and correctly classified 90.7% of the cases.
2. The model predicting the *Willingness to start or continue participating in the provision of lectures or skills-based teaching for psychiatry residents* was statistically significant; *Χ*^*2*^ (df=4; *n*=54) = 16.73, *p* < .01, accounting for 26.6% (Cox and Snell R^2^) to 43.2% (Nagelkerke R^2^) of the variance; and correctly classified 81.5% of the cases.
3. The model predicting the *Willingness to start or continue participating in skills-based examinations of psychiatry residents* was statistically significant; *Χ*^*2*^ (df=6; *n*=54) = 29.29, *p* < .001, accounting for 41.9% (Cox and Snell R^2^) to 65.8% (Nagelkerke R^2^) of the variance; and correctly classified 88.9% of the cases.
4. The model predicting the *Willingness to participate in training and supervision of Canadian-trained psychiatrists undertaking a one-year Dalhousie University accredited fellowship experience in area of expertise* was statistically significant; *Χ*^*2*^ (df=2; *n*=53) = 10.39, *p* < .01, accounting for 17.8% (Cox and Snell R^2^) to 29.8% (Nagelkerke R^2^) of the variance; and correctly classified 81.1% of the cases.
5. The model predicting the *Willingness to participate in the training and supervision of internationally trained psychiatrists seeking to undertake the Dalhousie University accredited Fellowships* was statistically significant; *Χ*^*2*^ (df=6; *n*=53) = 29.20, *p* < .001, accounting for 42.4% (Cox and Snell R^2^) to 70.8% (Nagelkerke R^2^) of the variance; and correctly classified 90.6% of the cases.

Table 3 summarizes the results from five logistic regression models aimed at predicting respondents' willingness to initiate or sustain participation in five specific teaching activities within the Department of Psychiatry at Dalhousie University's DME. Controlling for other variables (see Appendix), only one variable - availability to participate in formal training in the RCPSC/CBD training, emerged as a significant predictor for four out of the five teaching activities. The exception was training/supervising internationally trained psychiatrists.

**Table 3:** Significant predictor (s) from five multivariate logistic regression models for the respondents’ likelihood to report willingness to participate in five specific teaching activities

**Predictor(s)**	**Willingness to start or continue participating in:**														
	**Clinical training/supervision of psychiatry residents**			**Provision of lectures or skills-based teaching for psychiatry residents**			**Skills-based examinations of psychiatry residents**			**Training/supervision of Canadian-trained psychiatrists**			**Training/supervision of internationally trained psychiatrists**		
	**OR**	**95% CI**	***P* value**	**OR**	**95% CI**	***P* value**	**OR**	**95% CI**	***P* value**	**OR**	**95% CI**	***P* value**	**OR**	**95% CI**	***P* value**
**Availability to participate in formal training in the RCPSC/CBD**	14.67	1.37-156.89	**.03**	5.58	1.03-30.19	**.046**	16.96	1.20-238.98	**.04**	9.53	1.86-48.69	**<.01**	-	-	-

*C.I.* Confidence interval

*P* value: Significance at *p*≤ .05

## Discussion

To the best of our knowledge, this is the first study to explore the factors influencing psychiatrists' at distributed sites willingness to engage in teaching activities, a pivotal element for the growth of DME. It provides academic decision-makers with evidence-based findings that may contribute to making well-informed decisions about the expansion of DME while actively involving local psychiatrists in this transformative process.

The outcomes revealed a strong willingness among psychiatrists to participate in various teaching activities, including clinical training/supervision of psychiatry residents, delivering lectures, conducting skills-based teaching/examinations, and providing training/supervision to Canadian and internationally trained psychiatry fellows. The average positive response rate reached in our study (81.98%) significantly surpassed the participation rate (65%) reported for medical specialists in teaching activities, as per the CFPC/CMA/RCPSC National Physician Survey in 2004 [13]. Interestingly, this enthusiasm persists despite the majority lacking formal training in medical education. This finding challenges the conventional definition of a clinician-educator by the Canadian Royal College, which typically involves formal education in teaching [14]. This underscores the unique dedication of physicians to serve clients and society, blending specialized knowledge with principles of responsibility, selflessness, and wisdom [15]. Yet, amid these auspicious results, challenges persist. The study identified significant barriers preventing psychiatrists from active participation in teaching activities. The top impediments reported were the lack of protected time for teaching/training and inadequate financial incentives. Interestingly, these findings paradoxically resonate with existing literature. On one hand, they underscore the well-documented struggle physicians face in balancing clinical practice with academic duties [16–20]. On the other hand, they deviate from commonly recognized barriers such as lack of recognition, inadequate faculty development, unmotivated students, low interest in teaching, bureaucratic academic cultures, and suboptimal learning environments [21].

However, the study's most intriguing findings lie in the factors associated with, and those that can predict, psychiatrists' willingness to engage in teaching. The main factor associated with psychiatrists' willingness to participate in the five specific teaching activities investigated in this study was the availability to take the RCPSC/CBD training. This training has been implemented by the RCPSC to transform postgraduate medical education in Canada. CBD focuses on achieving competencies rather than traditional time-based training and emphasizing outcomes, such as the ability to apply medical knowledge, professionalism, communication, and collaboration skills [22].

The relationship between the RCPSC/CBD training and clinicians' willingness to be involved in teaching activities could be multifaceted, and it has not yet been reported in the literature. Nonetheless, individuals expressing interest in such training may show a preference for modern educational approaches, emphasizing practical skills and competency development, potentially indicating an initial inclination toward increased participation in teaching activities. Training psychiatrists with the necessary experience to continue providing outreach services in their practice within rural communities is one of the strategies to address the needs of underserviced areas from an educational perspective [23]. This result might emphasize the potential cascading effect of participation in structured training programs, even though the absence of training was not deemed a significant barrier for participants engaging in teaching activities.

The RCPSC/CBD training availability emerged as the sole predictor for psychiatrists' readiness to partake in four out of five teaching activities, excluding involvement in the training/supervision of internationally trained psychiatrists. This finding offers a straightforward and reliable method to identify psychiatrists at DME sites who are inclined to contribute to teaching efforts.

Other variables were found to be associated with the psychiatrists' willingness to be involved in teaching activities. Psychiatrists working in New Brunswick were more willing to engage in these activities compared to their counterparts in Nova Scotia. This finding suggests potential regional variations in the motivation and opportunities for psychiatrists to contribute to medical education. This finding supports the local needs of expanding the number of psychiatrists in New Brunswick region.

Having an academic appointment with the Department of Psychiatry at Dalhousie University was also significantly associated with the willingness to participate in the provision of lectures or skills-based teaching for psychiatry residents. This finding once more shed light on the association between academic affiliations on physicians' involvement in medical education. Psychiatrists with academic appointments often have access to resources, mentorship, and a supportive academic environment, which may foster their enthusiasm for teaching and supervision activities.

The practice of psychotherapy also emerged as a significant factor associated with psychiatrists' willingness to engage in the provision of lectures or skills-based teaching for psychiatry residents. Psychiatrists specializing in psychotherapy often develop strong interpersonal and communication skills, which are highly transferable to teaching and mentoring roles. These skills include active listening, empathy, and creating a supportive and collaborative therapeutic environment. In this context, the willingness of these psychiatrists to engage in teaching and mentoring activities might be attributed to their specific skills.

In addition to considering psychiatrists' willingness to engage in teaching activities, it is crucial to account for other factors that may impact the successful expansion of DME. Previous research demonstrate that early exposure to the field of psychiatry correlates with subsequent practice in rural and remote locations [24]. Future investigations could explore how expanded DME residency training and academic teaching opportunities can impact this decision in the future generations of medical students.

## Strengths and Limitations

The study has several strengths that contribute to the consistency of its findings. Firstly, it adopts a comprehensive approach by employing a rigorous data collection process and ensuring the questionnaire's relevance and validity through development and revision by members of the Nova Scotia and New Brunswick Psychiatry Academic Council. Additionally, conducting data collection during psychiatrists' meetings provides an organized and conducive environment for survey completion, further enhancing the reliability of the data. The study's sample is also a strength, as it includes psychiatrists from seven administrative health zones in Nova Scotia and New Brunswick, representing a diverse range of rural and urban settings.

However, it is important to acknowledge certain limitations of the study. The reliance on voluntary participation introduces the possibility of selection bias. Psychiatrists with a stronger interest in medical education or more pronounced opinions on the topic may have been more likely to participate, potentially leading to the overrepresentation or underrepresentation of certain perspectives. Additionally, the study relies on self-reported data, which can be susceptible to biases such as social desirability bias or recall bias [25]. Participants' responses may not always accurately reflect their actual behaviors or opinions. While the study includes a diverse sample of psychiatrists from different settings, the findings may not be fully generalizable to all psychiatrists in the Maritimes Provinces or other regions, as regional variations in healthcare systems and educational resources can influence the willingness to engage in teaching activities differently in different contexts. Lastly, although the study controls for various variables in the regression analyses, there may still be unmeasured factors that could confound the relationships between the predictors and outcome variables, potentially influencing the observed associations.

## Conclusions

This study provides actionable insights for academic decision-makers to improve psychiatry education at distributed medical education (DME) sites. By addressing identified barriers, recognizing regional variations, and understanding the impact of training programs and practice specialties, academic departments can strategically promote and facilitate the active engagement of psychiatrists in teaching activities. The study's outcomes underscore the paradoxical complexity of engaging clinicians in medical education. While the most significant factor associated with and predicting their willingness to engage in teaching activities was interest in formal medical education training, clinicians did not perceive the lack of formal training as a barrier. This duality presents a potential pathway for enhancing physician involvement or a pitfall if other factors, such as the lack of protected time and adequate financial incentives, are not addressed. To navigate this complexity, governments, health and academic institutions should consider targeted strategies to tackle these barriers, including resource allocation, policy changes, and incentive structures. Above all, fostering a culture that actively acknowledges and encourages psychiatrists' enthusiasm for teaching is paramount and may ensure a conducive environment for sustained and meaningful engagement at DME sites.

### Supplementary Information

**Supplementary Material 1.** 

**Supplementary Material 2.** 

**Supplementary Material 3.** 

## Data Availability

The datasets used and/or analysed during the current study are available from the corresponding author on reasonable request.

## Abbreviations

DME: Distributed Medical Education
NS: Nova Scotia
NB: New Brunswick
CME: Continued Medical Education
RCPSC: Royal College of Physicians and Surgeons of Canada
IMG: International Medical Graduates
CMG: Canadian Medical Graduates
CBT: Cognitive Behavioural Therapy
CBD: Competency by Design
