# Supplementary material for: Distributed Medical Education (DME) in psychiatry: perspectives on facilitators, obstacles, and factors affecting psychiatrists' willingness to engage in teaching activities
Source: BMC Med Educ. 2024 Feb 25;24:192. doi: 10.1186/s12909-024-05178-8 (PMC10895840; doi:10.1186/s12909-024-05178-8)
Supplement: Supplementary file 1 — Supplementary Material 1. [file 12909_2024_5178_MOESM1_ESM.docx]

**Appendix A**: Detailed regression models utilized

1. Logistic regression model predicting respondents’ willingness to start or continue participating in clinical training and supervision of psychiatry residents

|  | B | S.E. | Wald | df | Sig. | Exp(B) | 95% C.I.for EXP(B) | |
| --- | --- | --- | --- | --- | --- | --- | --- | --- |
|  |  |  |  |  |  |  | Lower | Upper |
| Do you practice psychotherapy? (Yes) | 19.046 | 8145.214 | .000 | 1 | .998 | 186970891.439 | .000 | . |
| Would you be available to participate in formal training in the RCPSC Competency by Design to facilitate your involvement in psychiatry residency training? (Yes) | 2.686 | 1.209 | 4.933 | 1 | .026 | 14.667 | 1.371 | 156.888 |
| Constant | .405 | .645 | .395 | 1 | .530 | 1.500 |  |  |

1. Logistic regression model predicting respondents’ willingness to start or continue participating in the provision of lectures or skills-based teaching for psychiatry residents

|  | B | S.E. | Wald | df | Sig. | Exp(B) | 95% C.I.for EXP(B) | |
| --- | --- | --- | --- | --- | --- | --- | --- | --- |
|  |  |  |  |  |  |  | Lower | Upper |
| Which province do you work in? (Nova Scotia) | -1.812 | 1.188 | 2.328 | 1 | .127 | .163 | .016 | 1.675 |
| Do you currently have an academic appointment with the Department of Psychiatry at Dalhousie University? (Yes) | 1.151 | .857 | 1.804 | 1 | .179 | 3.161 | .589 | 16.953 |
| Do you practice psychotherapy? (Yes) | 1.407 | 1.233 | 1.302 | 1 | .254 | 4.083 | .364 | 45.775 |
| Would you be available to participate in formal training in the RCPSC Competency by Design to facilitate your involvement in psychiatry residency training? (Yes) | 1.719 | .862 | 3.980 | 1 | .046 | 5.578 | 1.031 | 30.192 |
| Constant | .675 | 1.288 | .275 | 1 | .600 | 1.964 |  |  |

1. Logistic regression model predicting respondents’ willingness to Willingness to start or continue participating in skills-based examinations of psychiatry residents

|  | B | S.E. | Wald | df | Sig. | Exp(B) | 95% C.I.for EXP(B) | |
| --- | --- | --- | --- | --- | --- | --- | --- | --- |
|  |  |  |  |  |  |  | Lower | Upper |
| Which province do you work in? (Nova Scotia) | -19.829 | 4879.195 | .000 | 1 | .997 | .00 | .000 | . |
| Which type of medical graduate are you? (IMG) | 1.476 | 1.468 | 1.011 | 1 | .315 | 4.38 | .246 | 77.710 |
| Do you currently have an academic appointment with the Department of Psychiatry at Dalhousie University? (Yes) | -.094 | 1.095 | .007 | 1 | .932 | .91 | .106 | 7.791 |
| Do you practice psychotherapy? (Yes) | 20.662 | 4879.195 | .000 | 1 | .997 | 940230262.83 | .000 | . |
| Are you familiar with the RCPSC Competency by Design for residency training? (Yes) | 20.324 | 4879.195 | .000 | 1 | .997 | 671003905.25 | .000 | . |
| Would you be available to participate in formal training in the RCPSC Competency by Design to facilitate your involvement in psychiatry residency training? (Yes) | 2.831 | 1.350 | 4.399 | 1 | .036 | 16.96 | 1.204 | 238.977 |
| Constant | -2.433 | 2.537 | .920 | 1 | .338 | .09 |  |  |

1. Logistic regression model predicting respondents’ willingness to participate in training and supervision of Canadian-trained psychiatrists undertaking a one-year Dalhousie University accredited fellowship experience in area of expertise

|  | B | S.E. | Wald | df | Sig. | Exp(B) | 95% C.I.for EXP(B) | |
| --- | --- | --- | --- | --- | --- | --- | --- | --- |
|  |  |  |  |  |  |  | Lower | Upper |
| Which province do you work in? (Nova Scotia) | -1.032 | .925 | 1.244 | 1 | .265 | .356 | .058 | 2.185 |
| Would you be available to participate in formal training in the RCPSC Competency by Design to facilitate your involvement in psychiatry residency training? (Yes) | 2.254 | .832 | 7.332 | 1 | .007 | 9.526 | 1.864 | 48.692 |
| Constant | .884 | .885 | 1.000 | 1 | .317 | 2.422 |  |  |

1. Logistic regression model predicting respondents’ willingness to participate in the training and supervision of internationally trained psychiatrists seeking to undertake the Dalhousie University accredited Fellowships

|  | B | S.E. | Wald | df | Sig. | Exp(B) | 95% C.I.for EXP(B) | |
| --- | --- | --- | --- | --- | --- | --- | --- | --- |
|  |  |  |  |  |  |  | Lower | Upper |
| Which province do you work in? (Nova Scotia) | -19.339 | 5276.761 | .000 | 1 | .997 | .000 | .000 | . |
| Which type of specialist training did you complete? (International) | 19.339 | 5276.762 | .000 | 1 | .997 | 250597891.063 | .000 | . |
| What is your primary specialization or scope of practice? (General adult Psychiatry) |  |  | .000 | 3 | 1.000 |  |  |  |
| What is your primary specialization or scope of practice? (Child and Adolescent Psychiatry) | 20.542 | 9134.441 | .000 | 1 | .998 | 834380140.416 | .000 | . |
| What is your primary specialization or scope of practice? (Geriatric Psychiatry) | .000 | 1.701 | .000 | 1 | 1.000 | 1.000 | .036 | 28.039 |
| What is your primary specialization or scope of practice? (Other) | 19.107 | 19932.773 | .000 | 1 | .999 | 198657730.600 | .000 | . |
| Would you be available to participate in formal training in the RCPSC Competency by Design to facilitate your involvement in psychiatry residency training? (Yes) | 20.726 | 5276.761 | .000 | 1 | .997 | 1002391564 | .000 | . |
| Constant | -.693 | 1.350 | .264 | 1 | .608 | .500 |  |  |
