# Supplementary material for: Distributed Medical Education (DME) in psychiatry: perspectives on facilitators, obstacles, and factors affecting psychiatrists' willingness to engage in teaching activities
Source: BMC Med Educ. 2024 Feb 25;24:192. doi: 10.1186/s12909-024-05178-8 (PMC10895840; doi:10.1186/s12909-024-05178-8)
Supplement: Supplementary file 2 — Supplementary Material 2. [file 12909_2024_5178_MOESM2_ESM.docx]

**Table S1:** Variables presenting no statistical significance (p > .01) in the Chi square association with psychiatrists’ willingness to participate in teaching activities for psychiatry residents

| **Variables** | **Clinical training/supervision** | | | **Provision of lectures or skills-based teaching** | | | **Skills-based examinations** | | |
| --- | --- | --- | --- | --- | --- | --- | --- | --- | --- |
|  | **N (%)** | **Chi^2^ /Fisher Exact*** | **P value** | **N (%)** | **Chi^2^ /Fisher Exact*** | **P value** | **N (%)** | **Chi^2^ /Fisher Exact*** | **P value** |
| **Work-in horizon Health Zone**  HZ1 NB: Moncton/SE area  HZ2 NB: Fundy Shore and Saint John Area  HZ3 NB: Fredericton and River Valley Area  Eastern Zone NS  Northern Zone NS  Western Zone NS | 8 (100.0)  10 (100.0)  6 (85.7)  5 (100.0)  5 (83.3)  15 (83.3) | * | .56 | 8 (100.0)  10 (100.0)  6 (85.7)  3 (60.0)  5 (83.3)  12 (66.7) | * | .10 | 8 (100.0)  10 (100.0)  6 (85.7)  3 (60.0)  4 (66.7)  12 (66.7) | * | .09 |
| **Type of medical graduates**  IMG  CMG | 31 (88.6)  18 (94.7) | * | .65 | 27 (77.1)  17 (89.5) | 1.24 | .27 | 26 (74.3)  17 (89.5) | 1.75 | .19 |
| **Type of completed specialist training**  International specialist training  Canadian specialist training | 23 (88.5)  26 (92.9) | * | .66 | 20 (76.9)  24 (85.7) | 0.69 | .41 | 20 (76.9)  23 (82.1) | 0.23 | .63 |
| **Gender**  Woman  Man | 16 (94.1)  33 (89.2) | * | .99 | 14 (82.4)  30 (81.1) | 0.01 | .91 | 14 (82.4)  29 (78.4) | 0.11 | 0.74 |
| **Primary specialization or scope of practice**  General adult Psychiatry  Child and adolescent psychiatry  Geriatric Psychiatry  Other | 30 (88.2)  13 (92.9)  3 (100.0)  3 (100.0) | * | .99 | 28 (82.4)  10 (71.4)  3 (100.0)  3 (100.0) | * | .60 | 28 (82.4)  9 (64.3)  3 (100.0)  3 (100.0) | * | .42 |
| **Primary mode of payment for the delivered psychiatric services**  Salary with benefits (e.g.,  pension)  Fee for service  Sessional fees  Alternate Funding Plan  Other | 17 (94.4)  7 (77.8)  8 (100.0)  10 (83.3)  7 (100.0) | * | .44 | 16 (88.9)  6 (66.7)  7 (87.5)  9 (75.0)  6 (85.7) | * | .67 | 15 (83.3)  7 (77.8)  7 (87.5)  8 (66.7)  6 (85.7) | * | .81 |
| **Received any formal training in medical education**  Yes  No | 19 (90.5)  30 (90.9) | * | .99 | 16 (76.2)  28 (84.8) | 0.64 | .43 | 15 (71.4)  28 (84.8) | 1.43 | .23 |
| **Years of experience in clinical training or supervision of medical learners**  More than 10 years  6-10 years  3-5 years  1-2 years  0 years | 26 (89.7)  9 (100.0)  8 (88.9)  5 (100.0)  1 (50.0) | * | .33 | 25 (86.2)  7 (77.8)  7 (77.8)  4 (80.0)  1 (50.0) | * | .57 | 23 (79.3)  7 (77.8)  7 (77.8)  5 (100.0)  1 (50.0) | * | .62 |
