# Supplementary material for: Distributed Medical Education (DME) in psychiatry: perspectives on facilitators, obstacles, and factors affecting psychiatrists' willingness to engage in teaching activities
Source: BMC Med Educ. 2024 Feb 25;24:192. doi: 10.1186/s12909-024-05178-8 (PMC10895840; doi:10.1186/s12909-024-05178-8)
Supplement: Supplementary file 3 — Supplementary Material 3. [file 12909_2024_5178_MOESM3_ESM.docx]

**Table S2:** Variables presenting no statistical significance (p > .01) in the Chi square association with psychiatrists’ willingness to participate in training/supervision of fellows

| **Variables** | **Willingness to participate in training/supervision of Canadian-trained psychiatrists** | | | **Willingness to participate in the training/supervision of internationally trained psychiatrists** | | |
| --- | --- | --- | --- | --- | --- | --- |
|  | **N (%)** | **Chi^2^ value** | **P value** | **N (%)** | **Chi^2^ value** | **P value** |
| **Work-in province**  Nova Scotia  New Brunswick | 22 (75.9)  22 (91.7) | * | .16 | 22 (75.9)  22 (91.7) | * | .16 |
| **Work-in horizon Health Zone**  HZ1 NB: Moncton/SE area  HZ2 NB: Fundy Shore and Saint John Area  HZ3 NB: Fredericton and River Valley Area  Eastern Zone NS  Northern Zone NS  Western Zone NS | 6 (85.7)  10 (100.0)  6 (85.7)  4 (80.0)  4 (66.7)  14 (77.8) | * | .54 | 6 (85.7)  9 (90.0)  7 (100.0)  4 (80.0)  5 (83.3)  13 (72.2) | * | .74 |
| **Type of medical graduates**  IMG  CMG | 28 (82.4)  16 (84.2) | 0.03 | .86 | 29 (85.3)  15 (78.9) | 0.35 | .56 |
| **Type of completed specialist training**  International specialist training  Canadian specialist training | 22 (88.0)  22 (78.6) | * | .47 | 23 (92.0)  21 (75.0) | * | .15 |
| **Having an academic appointment with the Department of Psychiatry at Dalhousie University**  Yes  No | 31 (81.6)  13 (86.7) | 0.20 | .66 | 32 (84.2)  12 (80.0) | 0.14 | .71 |
| **Gender**  Woman  Man | 14 (82.4)  30 (83.3) | 0.01 | .93 | 13 (76.5)  31 (86.1) | 0.76 | .38 |
| **Primary specialization or scope of practice**  General adult Psychiatry  Child and adolescent psychiatry  Geriatric Psychiatry  Other | 25 (75.8)  13 (92.9)  3 (100.0)  3 (100.0) | * | .51 | 25 (75.8)  14 (100.0)  2 (66.7)  3 (100.0) | * | .13 |
| **Practicing psychotherapy**  Yes  No | 18 (90.0)  26 (78.8) | 1.11 | .29 | 18 (90.0)  26 (78.8) | 1.11 | .29 |
| **Primary mode of payment for the delivered psychiatric services**  Salary with benefits (e.g., pension)  Fee for service  Sessional fees  Alternate Funding Plan  Other | 15 (88.2)  7 (77.8)  6 (75.0)  9 (75.0)  7 (100.0) | * | .63 | 15 (88.2)  8 (88.9)  5 (62.5)  9 (75.0)  7 (100.0) | * | .35 |
| **Received any formal training in medical education**  Yes  No | 15 (75.0)  29 (87.9) | 1.47 | .23 | 15 (75.0)  29 (87.9) | 1.47 | .23 |
| **Years of experience in clinical training or supervision of medical learners**  More than 10 years  6-10 years  3-5 years  1-2 years  0 years | 22 (78.6)  8 (88.9)  8 (88.9)  5 (100.0)  1 (50.0) | * | .51 | 24 (85.7)  7 (77.8)  8 (88.9)  4 (80.0)  1 (50.0) | * | .62 |
| **Familiarity with the RCPSC Competency by Design for residency training**  Yes  No | 37 (86.0)  7 (70.0) | 1.48 | .22 | 36 (83.7)  8 (80.0) | 0.08 | .78 |

**IMG**: International Medical Graduate; **CMG**: Canadian Medical Graduate
